# Atg1 family kinases in autophagy initiation

**DOI:** 10.1007/s00018-015-1917-z

**Published:** 2015-05-07

**Authors:** Nobuo N. Noda, Yuko Fujioka

**Affiliations:** 1 Institute of Microbial Chemistry (BIKAKEN), 3-14-23 Kamiosaki, Shinagawa-ku, Tokyo, 141-0021 Japan; 2 CREST, JST, Tokyo, Japan

**Keywords:** Atg1 complex, ULK1 complex, Atg13, Atg17, FIP200, Kinase domain, MIT domain

## Abstract

Autophagosome formation, a landmark event in autophagy, is accomplished by the concerted actions of Atg proteins. Among all Atg proteins, Atg1 kinase in yeast and its counterpart in higher eukaryotes, ULK1 kinase, function as the most upstream factor in this process and mediate autophagy initiation. In this review, we summarize current knowledge of the structure, molecular function, and regulation of Atg1 family kinases in the initiation of autophagy.

## Introduction

Autophagy is an intracellular degradation system conserved among eukaryotes. In autophagy, a membrane structure named an isolation membrane appears suddenly in the cytoplasm, which then expands and becomes a double membrane-bound structure named an autophagosome [1, 2]. During this process, a portion of cytoplasm, including proteins and organelles, is sequestered into the autophagosome. The autophagosome then fuses with a lysosome (or a vacuole, in yeast and plants) and the inner membrane, which is named an autophagic body in yeast, is exposed to lysosomal hydrolases and degraded together with inner materials [1, 2]. The principal role of autophagy is to maintain cell homeostasis by recycling intracellular materials. Moreover, in higher eukaryotes such as mammals, autophagy mediates various physiological roles, and defects in this process are directly linked to severe diseases [3–6].

Studies using a species of budding yeast, *Saccharomyces cerevisiae*, identified 18 autophagy-related (Atg) proteins essential for autophagosome formation [2]. They are classified into 6 functional groups that include: the Atg8 and Atg12 conjugation systems, the autophagy-specific phosphatidylinositol 3-kinase (PI3K) complex, the Atg2–Atg18 complex, the transmembrane protein Atg9, and the Atg1 kinase complex, which are conserved from yeast to mammals, except for some regulatory components [2]. They are targeted to the pre-autophagosomal structure (PAS) in a hierarchical manner. Among six Atg groups, the Atg1 kinase complex functions as the most upstream factor [7–10]. Autophagy is induced strongly upon starvation, and starvation signals are thought to be transmitted initially to the Atg1 kinase complex [2, 11, 12]. Thus, understanding the function and regulation of the Atg1 kinase complex is essential for unveiling the molecular mechanism of autophagy initiation.

In this review, we summarize current knowledge of the structure and basic molecular functions of Atg1 family kinases, especially yeast Atg1 and its mammalian counterpart, Unc-51-like kinase 1 (ULK1), in autophagy initiation and discuss unsolved questions regarding these proteins. The physiological and medical roles of Atg1 family kinases as well as structural biological view on other Atg proteins are not mentioned here; thus, the reader should refer to the reviews published elsewhere [12–18].

## Components of the Atg1/ULK1 kinase complex

**Fig. 1:**
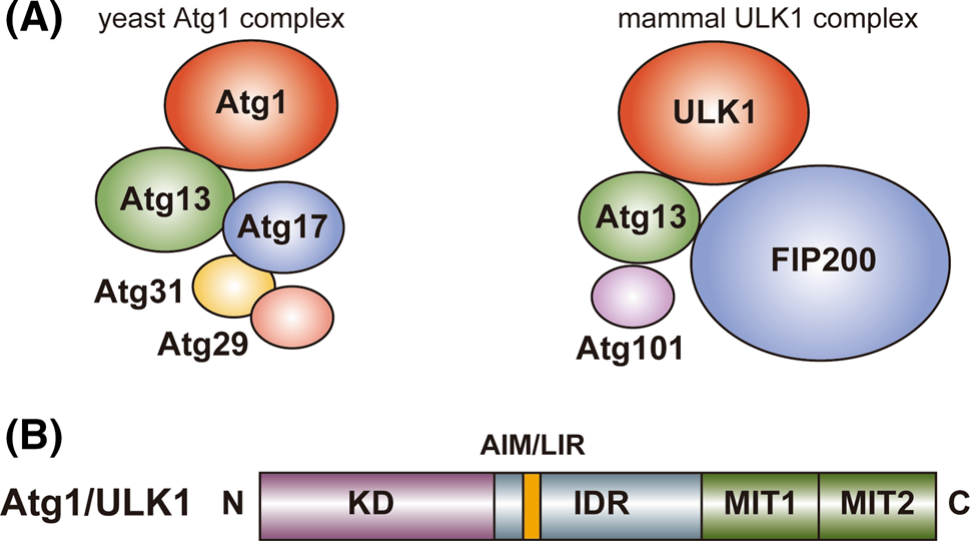
Schematic drawing of the domain organization and the binding partners of Atg1/ULK1. **a** Yeast (*S. cerevisiae*) Atg1 and mammalian ULK1 complexes. **b** Domain organization of Atg1/ULK1. *N* and *C* show amino- and carboxy-terminus, respectively

As a complex that is necessary for starvation-induced autophagy, *S. cerevisiae* Atg1 forms a pentameric complex with Atg13, Atg17, Atg29, and Atg31 [9, 11, 19], whereas mammalian ULK1 forms a tetrameric complex with Atg13, Atg101, and FIP200 also known as RB1CC1 (referred to as the ULK1 complex) (Fig. 1a) [20–26]. In mammals, a ULK1 paralog, ULK2, has functional redundancy with ULK1 in autophagy [27], but only the ULK1 complex is mentioned hereafter. In addition to starvation-induced autophagy, the ULK1 complex functions in selective types of autophagy such as mitophagy (selective autophagy of mitochondria), whereas the pentameric Atg1 complex seems to function specifically in starvation-induced autophagy [2]. In *S. cerevisiae*, selective autophagy such as the cytoplasm-to-vacuole targeting pathway is mediated by distinct types of Atg1 complexes whose components include Atg11 instead of Atg17, Atg29, and Atg31 [10, 28]. Since the pentameric Atg1 complex has been characterized more fully than the other types of Atg1 complexes involved in selective autophagy, only the pentameric Atg1 complex (simply referred to as the Atg1 complex) is mentioned hereafter.

## Architecture of Atg1 family kinases

Atg1 consists of 897 amino acids and possesses two globular domains, the serine/threonine kinase domain (KD) at the N-terminal region and two tandem microtubule-interacting and transport (MIT) domains at the C-terminal region (Fig. 1b) [29, 30]. The sequences of the KD and MIT are relatively highly conserved among Atg1 family kinases from yeast to mammals [31]. The region connecting the KD and MIT, which consists of approximately 250 and approximately 500 amino acids in Atg1 and ULK1, respectively, is less conserved and is predicted to be an intrinsically disordered region (IDR). Since this region of mammalian ULK1 is extremely abundant with Pro and Ser (both of which contribute about 17 % of the residues in this region), it was named the PS domain [31]. In the case of Atg1, Ser is abundant (about 14 % of the residues composing the IDR), but Pro is not (about 6 %).

## Architecture of the kinase domain

**Fig. 2:**
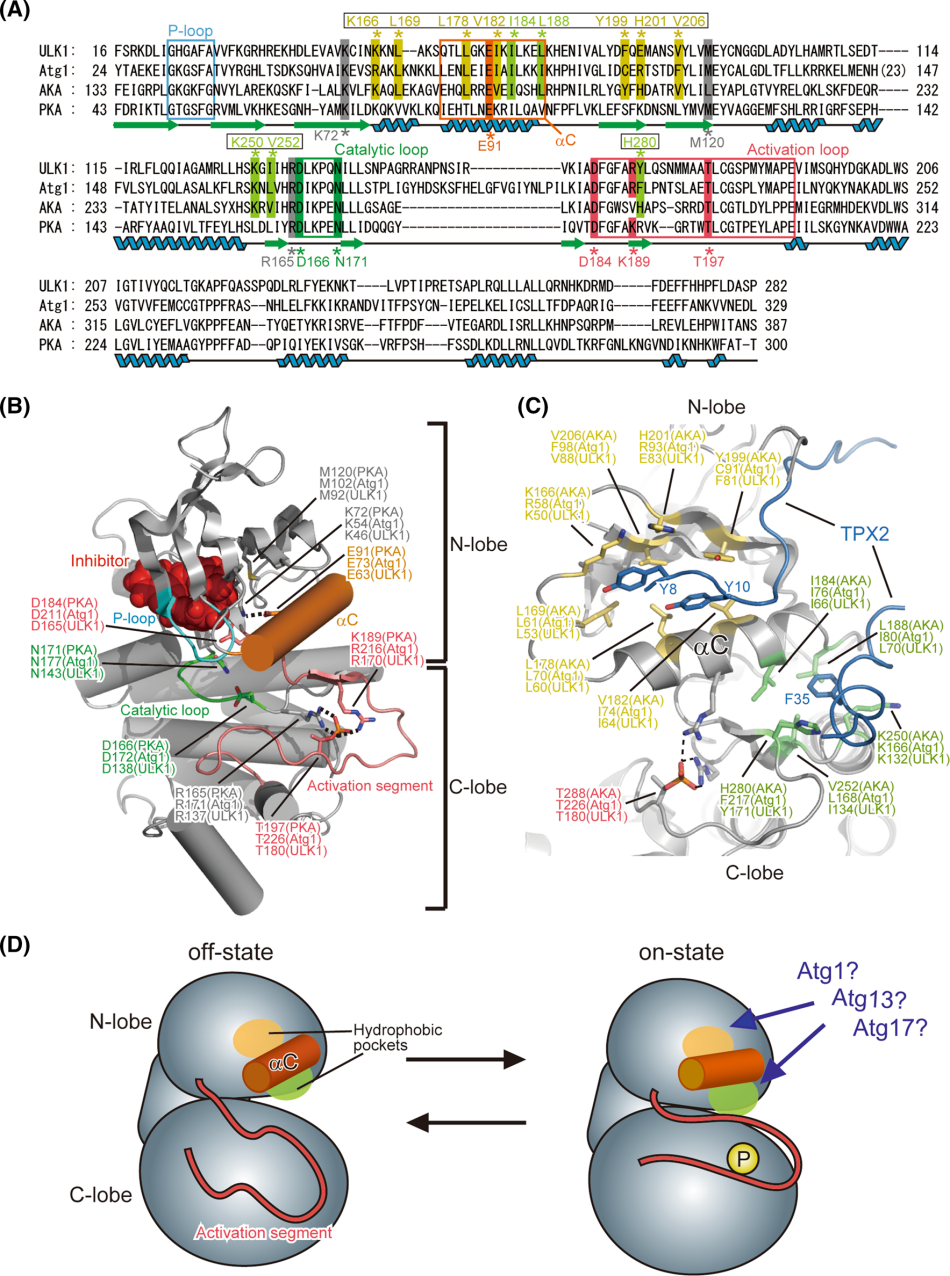
Proposed architecture of Atg1/ULK1 KD. **a** Sequence alignment among Atg1, ULK1, AKA and PKA. Secondary structure elements of PKA are denoted under the sequence. *Unboxed residue numbers* indicated below the sequence correspond to those of PKA, whereas *boxed residue numbers* indicated above the sequence correspond to those of AKA. **b** Crystal structure of the kinase domain of ULK1 (PDB ID 4WNO) [101]. The side chains of the catalytically important residues are shown with a *stick model*. *Broken lines* indicate possible salt bridges that are important for the on-state of the kinase. All structural models in this manuscript were prepared using the program PyMOL [102]. **c** Crystal structure of AKA in complex with TPX2 (PDB ID 1OL5). The side-chain of the residues that constitute the two hydrophobic pockets accommodating TPX2 is shown with a *stick model*. **d** Schematic drawing of the conformational switch of Atg1 KD between the off-state and on-state

The structure of the KD of human ULK1 has been determined by X-ray crystallography as a complex with its inhibitors [32]. ULK1 KD has a typical fold conserved among protein kinase superfamily. Among the structure-reported members of the protein kinase superfamily, the KD of Aurora kinase A (AKA) shows the highest sequence homology with Atg1 (31 % identity) and the KD of protein kinase A (PKA) has been most extensively characterized [33]. Figure 2a shows the sequence alignment of the KDs from Atg1, ULK1, AKA, and PKA. All eukaryote protein kinases conserve a fold similar to the KD of PKA and this is also true for ULK1 [34–38] (Fig. 2b). Furthermore, the PKA residues important for catalysis and regulation are also conserved in Atg1/ULK1 (Fig. 2a). Thus, the important residues and regions that have been characterized for PKA were mapped on the structure of ULK1 KD (Fig. 2b). The KD consists of two globular folds named the N- and C-lobe. The N-lobe mainly consists of a five-stranded β-sheet and an α-helix named αC (colored orange in Fig. 2b); whereas the C-lobe is mostly helical. All protein kinases utilize ATP for phosphorylating protein substrates. ATP binds to the cleft formed between both lobes and is covered by a lid named the P-loop or Gly-rich loop, which contains the highly conserved ATP-binding motif G-X-G-X-Φ-G, where Φ is usually Tyr or Phe [37]. In the case of Atg1 and ULK1, they both have the G-X-G-X-F-A sequence. Phosphorylation at Ser34 in the P-loop of Atg1, which was shown to impair its kinase activity [39], will block proper binding of ATP. The cleft between both lobes also accommodates the substrate, which comes into close contact with the *γ*-phosphate group of bound ATP and can thus be phosphorylated. A loop located between both lobes, named the activation segment (also known as the activation loop; colored pink in Fig. 2b), is involved in recognition of the substrate and regulation of kinase activity [34–37].

## Conformational switch of the kinase domain

KDs have at least two conformations, an on- and off-state, and the kinase activity of the former and latter is maximum and minimum, respectively [37]. Although the conformation of the off-state varies largely, especially at the activation segment, that of the on-state is highly conserved among kinases and this is also true for ULK1 [32, 34, 37]. The activation segment contains a phosphorylation site (Thr197 in PKA), whose phosphorylation generates salt bridges between the phosphate group and the side chains of Arg165 and Lys189 [38]. These interactions induce a conformational change of the activation segment as well as its surrounding regions such as αC, inducing a salt bridge between Glu91 in αC and Lys72 in the central β-sheet of the N-lobe. These changes switch the conformation of the KD into the on state [34, 37]. This on-state conformation was also observed in the crystal structure of ULK1: Thr180 of recombinant ULK1, which corresponds to Thr197 in PKA, was phosphorylated possibly via self-phosphorylation and formed salt-bridges with Arg137 and Arg170, and Glu63 in αC formed a salt-bridge with Lys46 in the central β-sheet of the N-lobe [32]. These similarities enabled researchers to design Atg1/ULK1 mutants based on the information obtained from other protein kinases; for example, mutation at Lys54 in Atg1 and Lys46 in ULK1, which are equivalent to Lys72 in PKA, was used to make inactive forms of Atg1/ULK1 [11, 26], and an alanine substitution at Met102 in Atg1, which is equivalent to the gatekeeper residue Met120 in PKA, was used to make an Atg1 mutant sensitive to the bulky ATP competitive inhibitor 1-NA-PP1, which does not inhibit wild-type protein kinases [40].

Proteomic analyses revealed that Thr226 of Atg1, which corresponds to Thr197 of PKA, was actually phosphorylated upon starvation, and its phosphorylation was shown to be essential for enhancing Atg1 kinase activity and inducing autophagy [41, 42]. Thr180 of ULK1 was also shown to be essential for ULK1 activation [43]. Atg13-binding reportedly induced an Atg1–Atg1 interaction and accelerated autophosphorylation at Thr226 of Atg1, thereby activating Atg1 [44]. However, phosphorylation at Thr226 is not sufficient to activate Atg1 and induce autophagy. Atg17 and FIP200 activate Atg1 and ULK1, respectively [24, 45]; however, the molecular mechanism remains to be addressed. AKA requires not only phosphorylation at Thr288 in the activation segment but also requires an interaction with TPX2 to switch the conformation to the on-state [33]. TPX2 mainly binds to the two hydrophobic pockets around αC: one is located between αC and the β-sheet of the N-lobe and the other is located between αC and the C-lobe (Fig. 2c). Both interactions are responsible for activating AKA by affecting the conformation of the activation segment and αC [33], and importantly, the residues constructing these hydrophobic pockets are conserved in Atg1 family kinases (Fig. 2a, c). Therefore, it could be speculated that Atg13 and/or Atg17 bind to these hydrophobic pockets similarly, thereby switching the conformation of Atg1 KD to the on-state (Fig. 2d). Alternatively, it could also be possible that intramolecular interactions switch the conformation of Atg1 to the on-state. This kind of intramolecular regulation is observed in various kinases such as PKA [36]. In the latter model, binding of Atg13 and/or Atg17 to the non-KD regions in Atg1 might induce a global conformational change of Atg1, which in turn might enable some portions of Atg1 to undergo intramolecular interactions with these hydrophobic pockets and thereby switch the conformation to the on-state. Further biochemical and structural studies on Atg1 family proteins are required to validate these speculations and establish the molecular mechanism of Atg1 kinase activation.

## Architecture of the MIT domains

**Fig. 3:**
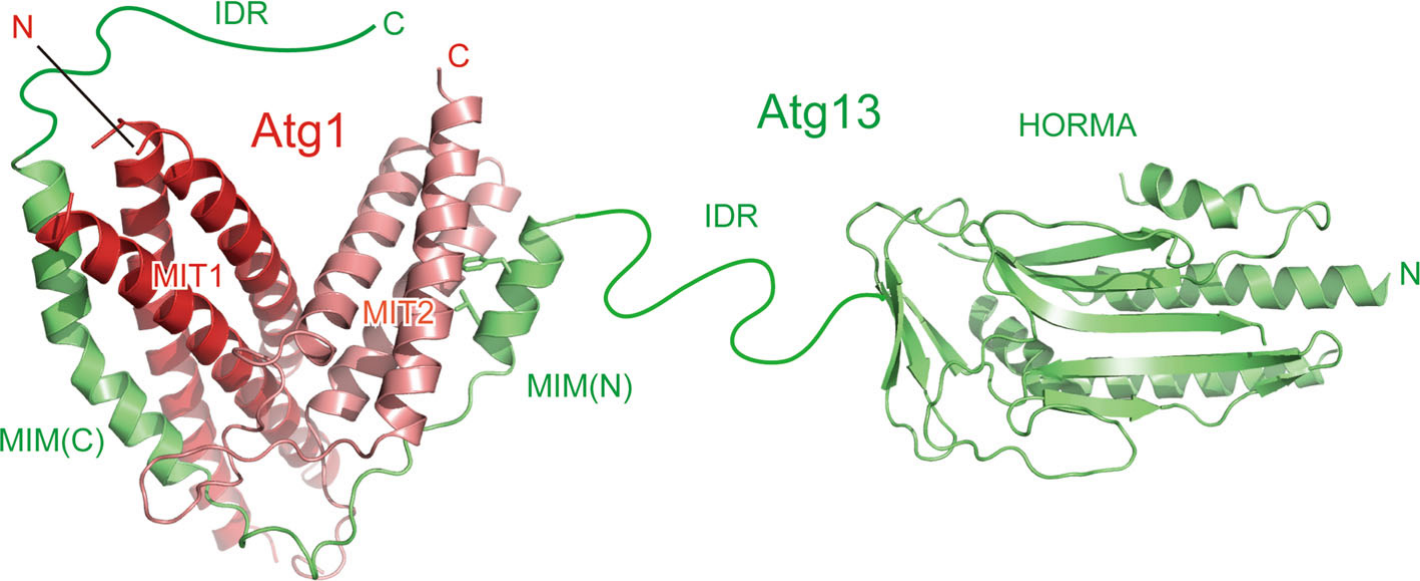
Architecture of Atg13 and tandem MIT domains of Atg1. Ribbon models are prepared using the crystal structures of Atg13 HORMA (PDB ID 4J2G) and two MIMs bound to the two MIT domains of Atg1 (PDB ID 4P1N). The side-chain of the two hydrophobic residues in MIM(N) that are important for Atg1 binding is shown with a *stick model*

Atg1 family kinases have a conserved globular domain at the C-terminal region, which is responsible for binding Atg13 [26, 44]. The crystal structure of the C-terminal region of Atg1 was determined as a complex with the Atg1-binding region of Atg13, which revealed that the C-terminal region consists of two MIT domains named MIT1 and MIT2 from the N terminus (Fig. 3) [29]. The MIT domain has a three-helix bundle architecture and is often observed in proteins involved in the multivesicular body pathway [46]. MIT domains normally function as a monomer and mediate protein–protein interactions [46]. In the case of Atg1, MIT1 and MIT2 interact with each other to form a single globular domain. This architecture is uncommon, but is also observed in Vta1, a protein involved in the multivesicular body pathway, in which two tandem MIT domains interact with each other to form a single globular domain [47]. A hydrogen–deuterium exchange coupled to mass spectrometry study suggested that MIT2 was flexible and unfolded partially in the absence of Atg13 and was folded and stabilized by Atg13 binding [48].

The C-terminal region of Atg1 family kinases reportedly interacts with membranes, especially highly curved membranes with a curvature radius of about 20 nm [26, 49]. However, the tandem MIT architecture of Atg1 does not possess a concave surface that is suitable for recognizing such curved membranes (Fig. 3). Recently, some MIT domains such as Vps4 have been reported to bind phosphoinositides in a manner dependent on calcium ions [50]. Two calcium-binding glutamates in Vps4 are not conserved in the equivalent region of the MIT domains of Atg1 family kinases and thus it is not clear whether they also bind calcium ions and phosphoinositides.

## Recognition of Atg13 by the MIT domains

MIT domains generally mediate protein–protein interactions and this is also true for Atg1 MIT domains, which directly recognize Atg13. Yeast Atg13 consists of 738 amino acids and possesses a globular domain named Hop1p, Rev7p, and Mad2 (HORMA) at the N-terminal region (Fig. 3) [51], which was recently shown to recruit Atg9 to the PAS via direct interaction [52]. The other C-terminal residues (about 450 residues) of Atg13 are responsible for Atg1 and Atg17 binding [11, 53]. They are predicted to be intrinsically disordered [29, 51], and therefore the C-terminal region is named IDR hereafter. MIT domains generally bind a helical region in target proteins, named the MIT-interacting motif (MIM), by forming an intermolecular helix bundle [46]. In the case of the Atg1–Atg13 interaction, a short α-helix of Atg13 named MIM(N) binds to the hydrophobic groove formed between the first and the third α-helices of Atg1^MIT2^, whereas another longer α-helix of Atg13 named MIM(C) binds to the hydrophobic groove formed between the second and the third α-helices of Atg1^MIT1^ (Fig. 3). The Atg1^MIT2^–Atg13^MIM(N)^ interaction is much stronger than the Atg1^MIT1^–Atg13^MIM(C)^ interaction in spite of the fact that the buried surface is larger for the latter interaction (Fig. 3) [29]. An alanine substitution at the hydrophobic residues in MIM(N) that are involved in binding Atg1^MIT2^ completely abolished the Atg1–Atg13 interaction in vivo, confirming that MIM(N) is the main binding site for Atg1 [29, 54]. Nevertheless, deletion of Atg13^MIM(C)^ markedly diminished the Atg1–Atg13 interaction in vivo [29], suggesting that MIM(C) plays a critical role in enhancing the Atg1–Atg13 interaction sufficiently for stable complex formation in vivo.

## Atg17, Atg29, and Atg31: yeast-specific components of the Atg1 complex

**Fig. 4:**
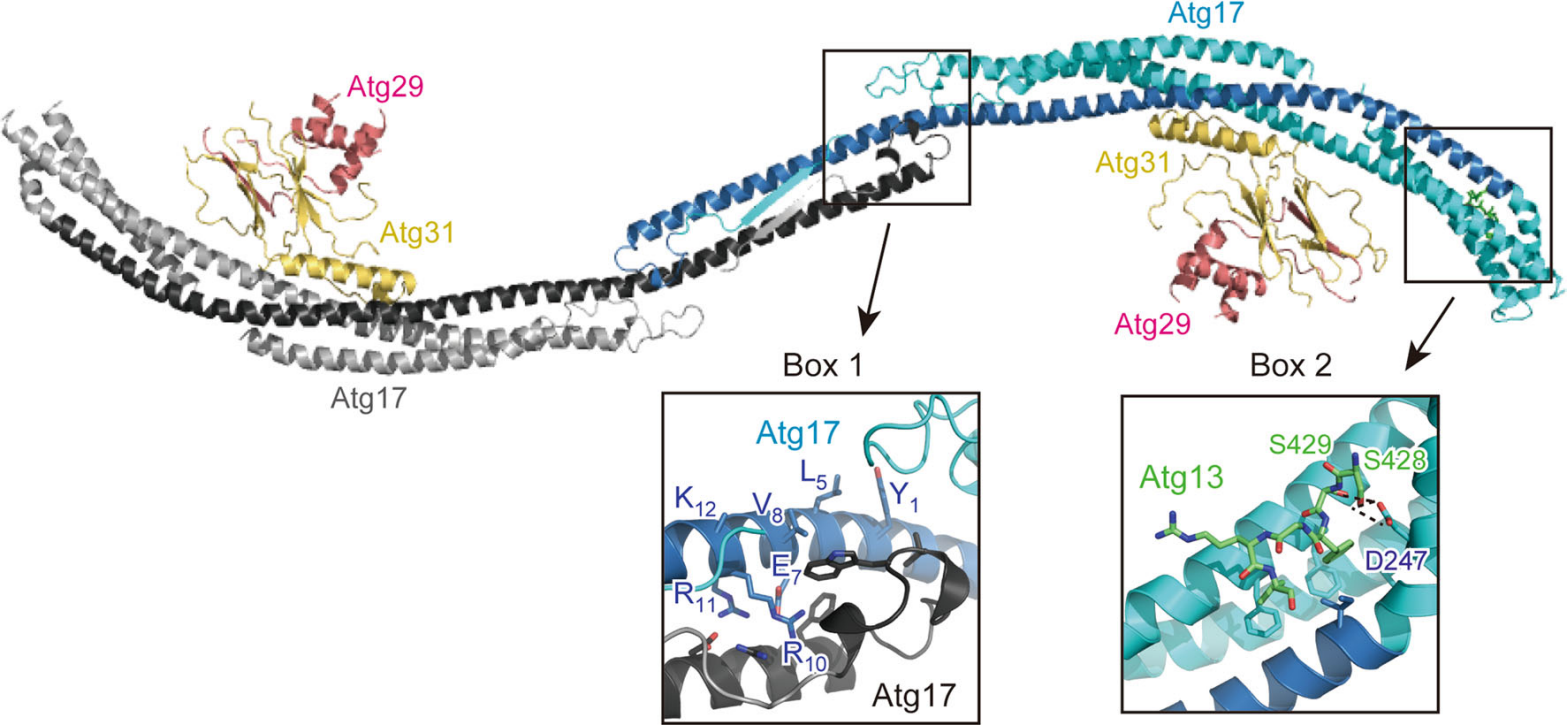
Architecture of the Atg17–Atg29–Atg31 complex and its interaction with Atg13^17BR^. Ribbon models were prepared using the crystal structure of the Atg13^17BR^–Atg17–Atg29–Atg31 complex (PDB ID 4P1W). Two protomers of Atg17 are colored *cyan* and *gray*, in which the regions that show weak homology with Atg11 and FIP200 are *colored blue* and *black*, respectively. *Boxes 1* and *2* indicate a close-up view of the dimer interphase and the 17BR-binding site, respectively

In addition to Atg1 and Atg13, the *S. cerevisiae* Atg1 complex possesses Atg17, Atg29, and Atg31 as essential components for autophagy although they are absent from higher eukaryotes such as mammals (Fig. 1a) [2, 9]. They constitutively form a ternary complex in vivo and form a stable complex with 2:2:2 stoichiometry in vitro [55]; thus, they are considered to be a single functional unit. The Atg17–Atg29–Atg31 complex interacts with Atg13 only under starvation conditions, which, together with the increased interaction between Atg1 and Atg13 under starvation, results in the formation of the pentameric Atg1 complex [11, 29, 45], although this model is currently controversial (discussed below) [54]. The direct interaction of the Atg17–Atg29–Atg31 complex with Atg1, if it exists, seems to be very weak since this interaction requires Atg13 in vivo [45]. Crystallographic and electron microscopic analyses established the unique architecture of the Atg17–Atg29–Atg31 complex (Fig. 4) [49, 56, 57]. Atg17 consists of four α-helices that fold into a crescent-like structure with a curvature radius of about 10 nm. Atg17 forms a stable homodimer using its C-terminal region, which results in a characteristic S-shape architecture. Atg31 binds directly to the concave surface of Atg17 using its C-terminal helix. Besides the C-terminal helix, Atg31 has an eight-stranded β-sandwich fold, in which one β-strand is derived from the N-terminal region of Atg29. Thus, Atg31 cannot maintain proper folding without Atg29. In addition to the N-terminal β-strand, Atg29 has a helical region and C-terminal IDR.

The main role of Atg17 is to function as a scaffold for the assembly of the PAS [8]. Atg17 interacts directly with a short region in the Atg13 IDR and this interaction has been revealed by crystallographic study on the complex formed between the Atg17–Atg29–Atg31 complex and an Atg13-derived peptide (Fig. 4) [29]. This interaction connects the Atg1–Atg13 complex to the Atg17–Atg29–Atg31 complex and the resultant pentameric Atg1 complex contributes to the construction of the PAS core. Atg9-containing vesicles, named Atg9 vesicles, are then targeted to the PAS [8, 58]. Since the radius of Atg9 vesicles (15–30 nm) is close to the curvature radius of the S-shape of Atg17 (about 10 nm), it was suggested that the S-shaped Atg17 dimer recognizes two Atg9 vesicles and thus tethers them to each other [49]. In this model, Atg29 and Atg31 located at the concave surface of Atg17 can be obstacles and they must be relocated before Atg9 vesicles can be accommodated. On the basis of these speculations, Atg29 and Atg31 were suggested to be negative regulators of autophagy initiation [49]. On the other hand, it has been established that both Atg29 and Atg31 are essential for autophagy [19, 59]. Interestingly, the S-shaped architecture of Atg17 was shown to be stabilized by binding Atg29 and Atg31 to the concave surface of Atg17, and without them, Atg17 showed an elongated conformation with less curvature [56]. Thus, the molecular roles and mechanisms of Atg17, Atg29, and Atg31 in Atg9 vesicle recognition are currently obscure. As for Atg29, it was shown that the C-terminal IDR of Atg29 has an inhibitory role in autophagy and multiple phosphorylation of the IDR cancels its inhibitory activity [57]. It was also demonstrated that Atg11, a scaffold protein essential for selective autophagy but not for starvation-induced autophagy [60], interacts directly with Atg29 IDR [57]. However, these observations cannot explain the essential role of Atg29 in starvation-induced autophagy. Thus, further structural and biochemical studies in parallel with cell biological studies are required for unveiling the molecular roles of these components in the Atg1 complex.

## FIP200 and Atg101: higher eukaryote-specific components of the ULK1 complex

In addition to ULK1 and Atg13, the mammalian ULK1 complex possesses FIP200 and Atg101 as essential components for autophagy although they are absent from *S. cerevisiae* (Fig. 1a) [12]. Although FIP200 has little sequence homology with Atg17 and is approximately four times larger than Atg17 (1594 versus 417 residues, respectively), it shares a common feature with Atg17, that is, possessing coiled-coils and interacting directly with Atg13. On the basis of these observations, FIP200 was suggested to be a functional counterpart of Atg17 [24]. On the other hand, another coiled-coil protein, Atg11, has significant sequence homology with FIP200 at the C-terminal region. Furthermore, careful sequence comparison of Atg11 and FIP200 with Atg17 suggested that approximately 150 residues in the N-terminal region of both Atg11 and FIP200 show weak sequence homology with Atg17, in which a consensus sequence, Y_1_-X_2_-X_3_-X_4_-(L/V/I)_5_-X_6_-E_7_-(V/I)_8_-X_9_-R_10_-R_11_-(R/K)_12_, is highly conserved [61]. The conserved 150-residue region corresponds to the central long α-helix of Atg17 (colored blue and black in Fig. 4), which forms a homo-dimer via the interaction mediated by the consensus sequence and other conserved residues (Fig. 4, box 1). Thus, FIP200 can be considered as a hybrid homolog of yeast Atg11 and Atg17 [12, 61]. Thus far, no structural information has been obtained experimentally for FIP200 and Atg11, and it is difficult to speculate their overall structure from their sequence. It is intriguing whether FIP200 and Atg11 possess a structural feature suitable for sensing membrane curvature as with the case of Atg17.

Atg101 was first identified as an Atg13-binding protein in mammals [23, 25]. Atg101 is conserved from mammals to fission yeast, but not in budding yeast [23]. Atg101 is essential for autophagy, but the only known function of Atg101 to date is to stabilize Atg13 [23, 25]. Atg101 binds to the N-terminal HORMA domain of Atg13 [23, 25], and interestingly, the structure of Atg101 was also predicted to be HORMA [62]. Therefore, the Atg101–Atg13 complex appears to be a heterodimeric HORMA. The HORMA protein Mad2 is known to switch between closed and open conformations, and the closed conformation recognizes its binding partner protein [63]. It remains to be established whether the Atg101–Atg13 heterodimeric HORMA has a specific binding partner(s), and if it exists, it is interesting whether their specific binding is regulated by conformational changes in their HORMA structure as discussed previously [51]. It is likely that species that conserve an Atg101 homolog do not conserve Atg29 and Atg31 homologs, whereas those that conserve Atg29 and Atg31 homologs do not conserve an Atg101 homolog [12, 61, 64]. This observation suggests that Atg101 and the Atg29–Atg31 pair undertake a similar role in autophagy. However, Atg101 has neither detectable sequence homology nor structural similarity with the Atg29–Atg31 pair. Furthermore, the binding partner of Atg101 is Atg13, whereas those of the Atg29–Atg31 pair are Atg17 and Atg11 [23, 25, 57]. Solving this riddle would break an impasse on understanding the molecular roles of the Atg1/ULK1 complex in autophagy.

## Interaction of Atg1 family kinases with Atg8 family proteins

Atg8 family proteins are ubiquitin-like proteins that are conjugated with a lipid, phosphatidylethanolamine (PE), through ubiquitin-like conjugation reactions [65, 66]. Atg8-PE localizes to autophagic membranes including the PAS, isolation membranes, complete autophagosomes, and autophagic bodies in the vacuole [7, 67]. Therefore, Atg8-PE can function as a scaffold on autophagic membranes and tethers selective cargoes to the membranes directly or through cargo receptors. Atg8-family proteins specifically recognize a (W/Y/F)_1_-X_2_-X_3_-(L/I/V)_4_ sequence, which is named the Atg8-family-interacting motif (AIM) or LC3-interacting region (LIR) [68, 69]. Atg1 family kinases conserve an AIM/LIR sequence in the IDR and interact directly with Atg8 family proteins, and thereby are tethered to isolation membranes [54, 70, 71]. This interaction delivers Atg1 family kinases to the vacuole in yeast and plants [54, 70, 72], whereas ULK1 seems to become detached from the isolation membrane together with Atg5 in mammals [73, 74]. Mutation at the AIM/LIR partially impairs autophagy, suggesting that localization of Atg1 family kinases to the isolation membrane promotes autophagy [54, 70]. However, it remains to be established whether Atg1 phosphorylates some factors and/or has a structural role on the isolation membrane. In vitro studies also reported that Atg13 and Atg17 possess AIM/LIR and bind Atg8-family proteins [71, 75]; however, their in vivo interaction and biological significance remain to be established.

## Regulation of the Atg1/ULK1 kinase complex by nutrient status

**Fig. 5:**
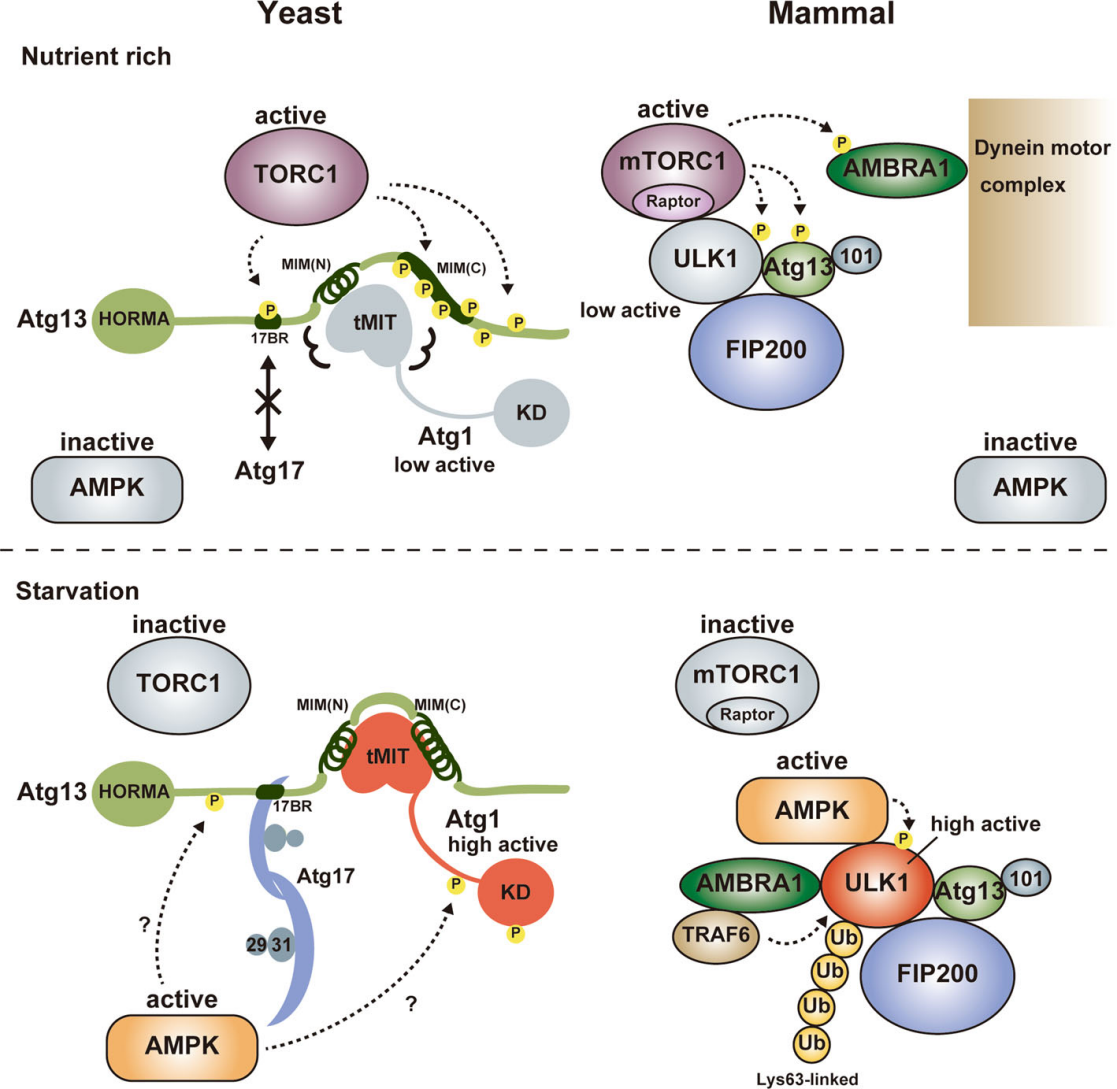
Summary of Atg1/ULK1 regulation. Under nutrient-rich conditions, TORC1 is active and directly phosphorylates Atg13 at 17BR and MIM(C), which impairs the formation of the Atg1 complex in yeast. In mammals, mTORC1 binds to ULK1 and phosphorylates ULK1, Atg13 and AMBRA1, which keeps the ULK1 complex as an inactive state although it does not impair the formation of the ULK1 complex. Upon starvation, TORC1 is inactive and Atg13 is dephosphorylated, which leads to the formation of the Atg1 complex in yeast. Activated AMPK also positively regulates the Atg1 complex possibly via phosphorylation of Atg1 and/or Atg13. In mammals, mTORC1 dissociates from the ULK1 complex and ULK1 is activated, which is also positively regulated by Lys63-linked ubiquitylation by the AMBRA1-TRAF6 complex and phosphorylation by activated AMPK. *Circled P* indicates phosphorylation sites

Since the Atg1/ULK1 kinase complex is the most upstream group of the six Atg groups involved in autophagosome formation, it functions as a hub to receive autophagy-initiating signals and transmits them to downstream Atg factors. TOR kinase complex 1 (TORC1) and AMP-activated protein kinase (AMPK) are major kinases that sense nutrient status and transmit it to the Atg1/ULK1 kinase complex. In addition to them, Ambra1 and PKA were reported as positive and negative regulators of autophagy in mammals and yeast through regulating ULK1 and Atg1, respectively [76–79]. Here, we focus on TORC1, AMPK and Ambra1 and summarize briefly the regulation of the Atg1/ULK1 kinase complex by them (Fig. 5).

### Regulation by TORC1

Nutrient starvation strongly induces autophagy in both yeast and mammals. It was known that rapamycin, a specific inhibitor of TORC1, induces autophagy similarly with starvation, indicating that TORC1 negatively regulates autophagy [79, 80]. In yeast, TORC1 directly phosphorylates Atg13 at multiple sites [81]. It was demonstrated that hyperphosphorylated Atg13 has only weak affinity with both Atg1 and Atg17 and the population of the Atg1 complex is low [11, 45]. When TORC1 is inhibited by starvation or treatment with rapamycin, Atg13 is dephosphorylated rapidly, which was suggested to increase its affinity for both Atg1 and Atg17 and enhance the population of the Atg1 complex, leading to an acute, dramatic induction of autophagy [11, 45]. However, a recent study proposed a conflicting model in which Atg1 and Atg13 form a stable complex regardless of nutrient conditions [54]. As mentioned above, Atg13 interacts with Atg1 using MIM(N) and MIM(C) (Fig. 3), whereas it interacts with Atg17 using a short region named 17BR (Fig. 4) [29]. MIM(N) has no phosphorylatable Ser/Thr residues, whereas MIM(C) has several Ser residues that are phosphorylated under nutrient-rich conditions and dephosphorylated upon starvation [29]. It was suggested that this phosphorylation reduces the affinity between Atg1 and Atg13 moderately, which leads to a great reduction of the Atg1–Atg13 complex population under starvation conditions [29]. However, MIM(N), the main binding site for Atg1, has no phosphorylation sites; thus, some population of the Atg1–Atg13 complex seems to exist even under nutrient-rich conditions. This might be one reason for the discrepant observations on the interaction between Atg1 and Atg13 [11, 54]. On the other hand, 17BR contains two Ser residues (Ser428 and Ser429) that are phosphorylated under nutrient-rich conditions and dephosphorylated upon starvation [29]. They form hydrogen bonds with Atg17 Asp247 (Fig. 4, box 2); therefore, phosphorylation of these Ser residues not only destroys essential hydrogen bonds but also causes electrostatic repulsion between the incorporated phosphate groups and the side-chain of Asp247. Thus, the interaction between Atg13 and Atg17 appears to be regulated more strictly by nutrient status than that between Atg1 and Atg13. Although TORC1 can be considered as the main kinase for phosphorylating these Ser residues, further studies are required to confirm the authentic kinase(s) for each phosphorylation site.

In the case of mammals, ULK1 forms a stable complex with Atg13, FIP200, and Atg101 irrespective of nutrient conditions [12, 20–22]. Although ULK1 and Atg13 are phosphorylated by mammalian TORC1 (mTORC1) under nutrient-rich conditions, it does not reduce the stability of the complex. It should be noted that FIP200 can be considered as a hybrid homolog of yeast Atg17 and Atg11 [12, 61], and that Atg11 interacts with Atg1 under nutrient-rich conditions [60]. This hybrid nature of FIP200 may be one reason for the constitutive formation of the ULK1 complex. mTORC1 interacts directly with ULK1 under nutrient-rich conditions and dissociates from it upon starvation, which was suggested to regulate the activity of the ULK1 complex [22]. This interaction is mediated by Raptor in mTORC1; however, the binding region in ULK1 for Raptor is controversial and it is uncertain whether the KD or IDR in ULK1 is the binding site for Raptor [22, 82]. Further biochemical and structural studies are required to establish whether mTORC1 inhibits the activity of ULK1 through a physical interaction and/or through phosphorylation of the components of the ULK1 complex.

### Regulation by AMPK

In addition to TORC1, AMPK is also a major regulator of starvation-induced autophagy in yeast and mammals [83–85]. In addition to the indirect regulation of autophagy by controlling TORC1 activity [86, 87], it was recently shown that AMPK regulates autophagy directly through phosphorylating and/or interacting with ULK1 in mammals [82, 88–90]. In contrast to TORC1, AMPK is inhibited under nutrient-rich conditions and activated upon starvation by sensing the accumulation of AMP. Several groups independently identified AMPK-mediated phosphorylation sites in the IDR of ULK1 and showed that their phosphorylation initiates autophagy through the activation of ULK1 [82, 88, 89]. Furthermore, the IDR of ULK1 was shown to be the starvation-dependent binding site for AMPK [82]. However, one group proposed a contradictory model in which AMPK inhibits ULK1 via a direct physical interaction under nutrient-rich conditions, and its dissociation from ULK1 upon starvation leads to the activation of ULK1 and initiation of autophagy [90]. Structural studies will be key to understand clearly the meaning of each phosphorylation by AMPK and the effect of AMPK binding on ULK1 activity.

### Regulation by AMBRA1

AMBRA1 was initially identified as a positive regulator of autophagy in vertebrates and was shown to promote the interaction between Beclin 1 and Vps34, two essential components of the PI3K complex [91]. Under nutrient-rich conditions, AMBRA1 is phosphorylated by mTORC1 and is anchored to the dynein motor complex, thereby being kept in an inactive state [92]. Upon starvation, activated AMBRA1 promotes Lys63-linked ubiquitylation of ULK1 via direct interaction with both ULK1 and an E3 ligase TRAF6, which leads to self-association, stabilization, and kinase-activity enhancement of ULK1 and initiation of autophagy [92]. Identification of ubiquitylation site(s) in ULK1 and characterization of the ubiquitylation effect on the ULK1 structure will be helpful for establishing the molecular mechanisms of ULK1 regulation by ubiquitylation.

## Molecular role of the Atg1/ULK1 kinase complex in autophagy initiation

**Fig. 6:**
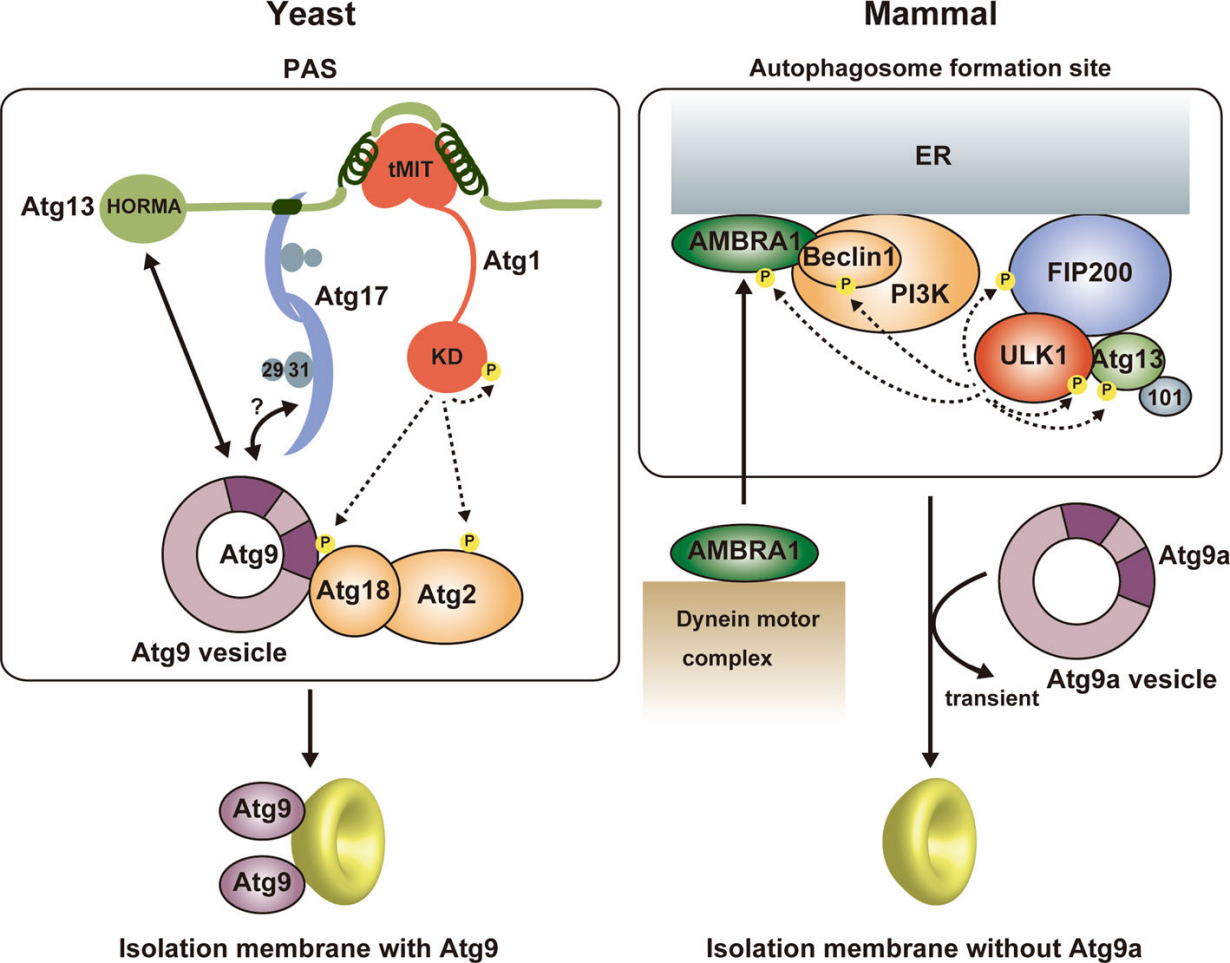
Summary of autophagy initiation mediated by Atg1/ULK1. In yeast, Atg9 vesicle is recruited to the PAS via the interaction with Atg13 HORMA and the Atg17–Atg9 interaction might also be responsible for that. Activated Atg1 phosphorylates Atg9, which promotes the recruitment of downstream factors such as Atg18 to the PAS. Atg9 is integrated into the isolation membrane. In mammals, activated ULK1 phosphorylates many factors, among which phosphorylation of Beclin 1 and AMBRA1 is responsible for the targeting of the ULK1 complex and the PI3 K complex to the autophagosome formation site at ER. In contrast to yeast Atg9, mammalian Atg9a targets to the autophagosome formation site independently of other Atg factors and transiently, and is not integrated into the isolation membrane

Among the six functional groups involved in autophagosome formation, the Atg1/ULK1 complex is the most upstream factor localized to the PAS in yeast and to the autophagosome formation site in mammals and plays a critical role in autophagy initiation (Fig. 6) [8, 93]. In yeast, a few Atg9 vesicles are localized to the PAS depending on the Atg1 complex and become an initial membrane source for isolation membranes [58]. Therefore, one molecular role of the Atg1 complex is the recruitment of Atg9 vesicles to the PAS. Although Atg17 reportedly binds Atg9, this interaction was dependent on Atg1 [94], and Atg13 was recently shown to directly bind Atg9 using the HORMA domain [52, 94]. As mentioned above, Atg17 has an S-shaped architecture whose concave surface seems to be suitable for recognizing Atg9 vesicles (Fig. 4) [49]. On the basis of that, an attractive hypothesis can be speculated: Atg17 tethers two Atg9 vesicles to each other using its S-shaped architecture, which leads to the fusion of Atg9 vesicles into early isolation membranes [49]. However, there is no experimental evidence supporting the recognition of Atg9 vesicles by the S-shaped architecture of Atg17. To complicate matters further, these relationships between the Atg1 complex and Atg9 observed in yeast are totally different from those in mammals. In contrast to yeast, mammalian Atg9a is targeted to the autophagosome formation site in a manner independent of the ULK1 complex [95]. Moreover, Atg9a only interacts transiently with the autophagosome formation site and is not incorporated into the isolation membrane [96]. These discrepancies between yeast and mammals are critical issues that must be elucidated prior to establishing the basic molecular mechanism of autophagy initiation.

The kinase activity of Atg1 family proteins is also essential for autophagy initiation [11, 24, 26, 45]. It seems to be evolutionarily conserved that the kinase activity of Atg1 family kinases is up-regulated upon starvation and by interaction with Atg13 and Atg17/FIP200 [11, 12, 24]. The kinase activity of Atg1 is known to be dispensable for targeting Atg proteins such as the Atg1 complex, Atg8, and Atg9 to the PAS [10, 94], whereas the kinase activity of ULK1 is required for targeting LC3 and Atg16L1 to the autophagosome formation site [24, 26]. Many substrates for Atg1 family kinases have been identified, which include the components of the Atg1/ULK1 complex such as Atg13, FIP200, and Atg1/ULK1 itself [21, 22, 26, 42, 97]. As mentioned above, autophosphorylation of Atg1/ULK1 is necessary for activation of its kinase activity [41–44], whereas the role of Atg13 and FIP200 phosphorylation by Atg1/ULK1 in autophagy remains to be established. Recently, screening for Atg1 kinase substrates using consensus peptide arrays identified Atg1, Atg2, and Atg9 as Atg1 substrates [98]. Furthermore, analyses using phosphorylation-mimic mutants suggested that phosphorylation of Atg9 by Atg1 is dispensable for proper Atg9 localization, but is crucial for efficient recruitment of Atg8 and Atg18 to the PAS through enhancing the Atg9–Atg18 interaction [98]. In mammals, Beclin 1 in an Atg14L-containing PI3K complex was reported to be phosphorylated directly by ULK1, which was shown to be required for the full induction of autophagy [99]. AMBRA1 was also shown to be phosphorylated by ULK1, which was suggested to release AMBRA1 from the dynein complex and target AMBRA1 to the ER together with other components of the PI3K complex to initiate autophagy [100]. However, the mechanism of how each phosphorylation affects the function of target proteins remains to be elucidated, and structural studies will be key to establish each mechanism at the molecular level.

## Concluding remarks

Recent studies identified many phosphorylation sites in Atg1/ULK1 and its activator Atg13 as well as in the substrates of Atg1/ULK1. Furthermore, structural studies on the Atg1 kinase complex have advanced markedly recently. These pieces of molecular information are now accelerating studies on the molecular roles of Atg1/ULK1 family kinases in autophagy initiation. However, it is still extremely difficult to elucidate the meaning of each phosphorylation at the molecular level since we have structural information on neither the IDRs of the Atg1/ULK1 kinase complex, which contain most of the important phosphorylation sites, nor the Atg1/ULK1 kinase substrates, such as Atg9 and the N-terminal domain of Beclin 1. To accelerate further studies on Atg1/ULK1 family kinases, it is critically important to perform cell biological and biochemical studies in parallel with structural studies.

## Abbreviations

AIM: Atg8-family interacting motif
AKA: Aurora kinase A
AMPK: AMP-activated protein kinase
HORMA: Hop1p, Rev7p, and Mad2
IDR: Intrinsically disordered region
KD: Kinase domain
LIR: LC3-interacting region
MIM: MIT-interacting motif
MIT: Microtubule interacting and transport
mTORC1: Mammalian TORC1
PAS: Pre-autophagosomal structure
PE: Phosphatidylethanolamine
PI3K: Phosphatidylinositol 3-kinase
PKA: Protein kinase A
TORC1: TOR kinase complex 1
ULK1: Unc-51-like kinase 1

## References

[1] Klionsky DJ, Ohsumi Y (1999). Vacuolar import of proteins and organelles from the cytoplasm. Annu Rev Cell Dev Biol.

[2] Mizushima N, Yoshimori T, Ohsumi Y (2011). The role of Atg proteins in autophagosome formation. Annu Rev Cell Dev Biol.

[3] Mizushima N, Levine B, Cuervo AM, Klionsky DJ (2008). Autophagy fights disease through cellular self-digestion. Nature.

[4] Jiang P, Mizushima N (2014). Autophagy and human diseases. Cell Res.

[5] Mizushima N, Komatsu M (2011). Autophagy: renovation of cells and tissues. Cell.

[6] Levine B, Mizushima N, Virgin HW (2011). Autophagy in immunity and inflammation. Nature.

[7] Suzuki K, Kirisako T, Kamada Y, Mizushima N, Noda T, Ohsumi Y (2001). The pre-autophagosomal structure organized by concerted functions of APG genes is essential for autophagosome formation. EMBO J.

[8] Suzuki K, Kubota Y, Sekito T, Ohsumi Y (2007). Hierarchy of Atg proteins in pre-autophagosomal structure organization. Genes Cells.

[9] Kawamata T, Kamada Y, Kabeya Y, Sekito T, Ohsumi Y (2008). Organization of the pre-autophagosomal structure responsible for autophagosome formation. Mol Biol Cell.

[10] Cheong H, Nair U, Geng J, Klionsky DJ (2008). The Atg1 kinase complex is involved in the regulation of protein recruitment to initiate sequestering vesicle formation for nonspecific autophagy in Saccharomyces cerevisiae. Mol Biol Cell.

[11] Kamada Y, Funakoshi T, Shintani T, Nagano K, Ohsumi M, Ohsumi Y (2000). Tor-mediated induction of autophagy via an Apg1 protein kinase complex. J Cell Biol.

[12] Mizushima N (2010). The role of the Atg1/ULK1 complex in autophagy regulation. Curr Opin Cell Biol.

[13] Wong PM, Puente C, Ganley IG, Jiang X (2013). The ULK1 complex: sensing nutrient signals for autophagy activation. Autophagy.

[14] Alers S, Wesselborg S, Stork B (2014). ATG13: just a companion, or an executor of the autophagic program?. Autophagy.

[15] Klionsky DJ, Schulman BA (2014). Dynamic regulation of macroautophagy by distinctive ubiquitin-like proteins. Nat Struct Mol Biol.

[16] Hurley JH, Schulman BA (2014). Atomistic autophagy: the structures of cellular self-digestion. Cell.

[17] Noda NN, Ohsumi Y, Inagaki F (2009). ATG systems from the protein structural point of view. Chem Rev.

[18] Noda NN, Inagaki F Mechanisms of autophagy. Annu Rev Biophys **in press**10.1146/annurev-biophys-060414-034248 25747593

[19] Kabeya Y, Kawamata T, Suzuki K, Ohsumi Y (2007). Cis1/Atg31 is required for autophagosome formation in Saccharomyces cerevisiae. Biochem Biophys Res Commun.

[20] Ganley IG, du Lam H, Wang J, Ding X, Chen S, Jiang X (2009). ULK1.ATG13.FIP200 complex mediates mTOR signaling and is essential for autophagy. J Biol Chem.

[21] Jung CH, Jun CB, Ro SH, Kim YM, Otto NM, Cao J, Kundu M, Kim DH (2009). ULK-Atg13-FIP200 complexes mediate mTOR signaling to the autophagy machinery. Mol Biol Cell.

[22] Hosokawa N, Hara T, Kaizuka T, Kishi C, Takamura A, Miura Y, Iemura S, Natsume T, Takehana K, Yamada N, Guan JL, Oshiro N, Mizushima N (2009). Nutrient-dependent mTORC1 association with the ULK1-Atg13-FIP200 complex required for autophagy. Mol Biol Cell.

[23] Hosokawa N, Sasaki T, Iemura S, Natsume T, Hara T, Mizushima N (2009). Atg101, a novel mammalian autophagy protein interacting with Atg13. Autophagy.

[24] Hara T, Takamura A, Kishi C, Iemura S, Natsume T, Guan JL, Mizushima N (2008). FIP200, a ULK-interacting protein, is required for autophagosome formation in mammalian cells. J Cell Biol.

[25] Mercer CA, Kaliappan A, Dennis PB (2009). A novel, human Atg13 binding protein, Atg101, interacts with ULK1 and is essential for macroautophagy. Autophagy.

[26] Chan EY, Longatti A, McKnight NC, Tooze SA (2009). Kinase-inactivated ULK proteins inhibit autophagy via their conserved C-terminal domains using an Atg13-independent mechanism. Mol Cell Biol.

[27] McAlpine F, Williamson LE, Tooze SA, Chan EY (2013). Regulation of nutrient-sensitive autophagy by uncoordinated 51-like kinases 1 and 2. Autophagy.

[28] Lynch-Day MA, Klionsky DJ (2010). The Cvt pathway as a model for selective autophagy. FEBS Lett.

[29] Fujioka Y, Suzuki SW, Yamamoto H, Kondo-Kakuta C, Kimura Y, Hirano H, Akada R, Inagaki F, Ohsumi Y, Noda NN (2014). Structural basis of starvation-induced assembly of the autophagy initiation complex. Nat Struct Mol Biol.

[30] Matsuura A, Tsukada M, Wada Y, Ohsumi Y (1997). Apg1p, a novel protein kinase required for the autophagic process in Saccharomyces cerevisiae. Gene.

[31] Yan J, Kuroyanagi H, Kuroiwa A, Matsuda Y, Tokumitsu H, Tomoda T, Shirasawa T, Muramatsu M (1998). Identification of mouse ULK1, a novel protein kinase structurally related to C. elegans UNC-51. Biochem Biophys Res Commun.

[32] Lazarus MB, Novotny CJ, Shokat KM (2015). Structure of the human autophagy initiating kinase ULK1 in complex with potent inhibitors. ACS Chem Biol.

[33] Bayliss R, Sardon T, Vernos I, Conti E (2003). Structural basis of Aurora-A activation by TPX2 at the mitotic spindle. Mol Cell.

[34] Taylor SS, Kornev AP (2011). Protein kinases: evolution of dynamic regulatory proteins. Trends Biochem Sci.

[35] Nolen B, Taylor S, Ghosh G (2004). Regulation of protein kinases; controlling activity through activation segment conformation. Mol Cell.

[36] Jura N, Zhang X, Endres NF, Seeliger MA, Schindler T, Kuriyan J (2011). Catalytic control in the EGF receptor and its connection to general kinase regulatory mechanisms. Mol Cell.

[37] Huse M, Kuriyan J (2002). The conformational plasticity of protein kinases. Cell.

[38] Knighton DR, Zheng JH, Ten Eyck LF, Ashford VA, Xuong NH, Taylor SS, Sowadski JM (1991). Crystal structure of the catalytic subunit of cyclic adenosine monophosphate-dependent protein kinase. Science.

[39] Yeh YY, Shah KH, Chou CC, Hsiao HH, Wrasman KM, Stephan JS, Stamatakos D, Khoo KH, Herman PK (2011). The identification and analysis of phosphorylation sites on the Atg1 protein kinase. Autophagy.

[40] Abeliovich H, Zhang C, Dunn WA, Shokat KM, Klionsky DJ (2003). Chemical genetic analysis of Apg1 reveals a non-kinase role in the induction of autophagy. Mol Biol Cell.

[41] Kijanska M, Dohnal I, Reiter W, Kaspar S, Stoffel I, Ammerer G, Kraft C, Peter M (2010). Activation of Atg1 kinase in autophagy by regulated phosphorylation. Autophagy.

[42] Yeh YY, Wrasman K, Herman PK (2010). Autophosphorylation within the Atg1 activation loop is required for both kinase activity and the induction of autophagy in *Saccharomyces cerevisiae*. Genetics.

[43] Bach M, Larance M, James DE, Ramm G (2011). The serine/threonine kinase ULK1 is a target of multiple phosphorylation events. Biochem J.

[44] Yeh YY, Shah KH, Herman PK (2011). An Atg13 protein-mediated self-association of the Atg1 protein kinase is important for the induction of autophagy. J Biol Chem.

[45] Kabeya Y, Kamada Y, Baba M, Takikawa H, Sasaki M, Ohsumi Y (2005). Atg17 functions in cooperation with Atg1 and Atg13 in yeast autophagy. Mol Biol Cell.

[46] Hurley JH, Yang D (2008). MIT domainia. Dev Cell.

[47] Xiao J, Xia H, Zhou J, Azmi IF, Davies BA, Katzmann DJ, Xu Z (2008). Structural basis of Vta1 function in the multivesicular body sorting pathway. Dev Cell.

[48] Stjepanovic G, Davies CW, Stanley RE, Ragusa MJ, Kim DJ, Hurley JH (2014). Assembly and dynamics of the autophagy-initiating Atg1 complex. Proc Natl Acad Sci USA.

[49] Ragusa MJ, Stanley RE, Hurley JH (2012). Architecture of the Atg17 complex as a scaffold for autophagosome biogenesis. Cell.

[50] Iwaya N, Takasu H, Goda N, Shirakawa M, Tanaka T, Hamada D, Hiroaki H (2013). MIT domain of Vps4 is a Ca^2+^-dependent phosphoinositide-binding domain. J Biochem.

[51] Jao CC, Ragusa MJ, Stanley RE, Hurley JH (2013). A HORMA domain in Atg13 mediates PI 3-kinase recruitment in autophagy. Proc Natl Acad Sci USA.

[52] Suzuki SW, Yamamoto H, Oikawa Y, Kondo-Kakuta C, Kimura Y, Hirano H, Ohsumi Y (2015). Atg13 HORMA domain recruits Atg9 vesicles during autophagosome formation. Proc Natl Acad Sci USA.

[53] Cheong H, Yorimitsu T, Reggiori F, Legakis JE, Wang CW, Klionsky DJ (2005). Atg17 regulates the magnitude of the autophagic response. Mol Biol Cell.

[54] Kraft C, Kijanska M, Kalie E, Siergiejuk E, Lee SS, Semplicio G, Stoffel I, Brezovich A, Verma M, Hansmann I, Ammerer G, Hofmann K, Tooze S, Peter M (2012). Binding of the Atg1/ULK1 kinase to the ubiquitin-like protein Atg8 regulates autophagy. EMBO J.

[55] Kabeya Y, Noda NN, Fujioka Y, Suzuki K, Inagaki F, Ohsumi Y (2009). Characterization of the Atg17–Atg29–Atg31 complex specifically required for starvation-induced autophagy in Saccharomyces cerevisiae. Biochem Biophys Res Commun.

[56] Chew LH, Setiaputra D, Klionsky DJ, Yip CK (2013). Structural characterization of the Saccharomyces cerevisiae autophagy regulatory complex Atg17–Atg31–Atg29. Autophagy.

[57] Mao K, Chew LH, Inoue-Aono Y, Cheong H, Nair U, Popelka H, Yip CK, Klionsky DJ (2013). Atg29 phosphorylation regulates coordination of the Atg17–Atg31–Atg29 complex with the Atg11 scaffold during autophagy initiation. Proc Natl Acad Sci USA.

[58] Yamamoto H, Kakuta S, Watanabe TM, Kitamura A, Sekito T, Kondo-Kakuta C, Ichikawa R, Kinjo M, Ohsumi Y (2012). Atg9 vesicles are an important membrane source during early steps of autophagosome formation. J Cell Biol.

[59] Kawamata T, Kamada Y, Suzuki K, Kuboshima N, Akimatsu H, Ota S, Ohsumi M, Ohsumi Y (2005). Characterization of a novel autophagy-specific gene, ATG29. Biochem Biophys Res Commun.

[60] Kim J, Kamada Y, Stromhaug PE, Guan J, Hefner-Gravink A, Baba M, Scott SV, Ohsumi Y, Dunn WA, Klionsky DJ (2001). Cvt9/Gsa9 functions in sequestering selective cytosolic cargo destined for the vacuole. J Cell Biol.

[61] Li F, Chung T, Vierstra RD (2014). AUTOPHAGY-RELATED11 plays a critical role in general autophagy- and senescence-induced mitophagy in Arabidopsis. Plant Cell.

[62] Hegedus K, Nagy P, Gaspari Z, Juhasz G (2014). The putative HORMA domain protein Atg101 dimerizes and is required for starvation-induced and selective autophagy in *Drosophila*. Biomed Res Int.

[63] Luo X, Yu H (2008). Protein metamorphosis: the two-state behavior of Mad2. Structure.

[64] Sun LL, Li M, Suo F, Liu XM, Shen EZ, Yang B, Dong MQ, He WZ, Du LL (2013). Global analysis of fission yeast mating genes reveals new autophagy factors. PLoS Genet.

[65] Ichimura Y, Kirisako T, Takao T, Satomi Y, Shimonishi Y, Ishihara N, Mizushima N, Tanida I, Kominami E, Ohsumi M, Noda T, Ohsumi Y (2000). A ubiquitin-like system mediates protein lipidation. Nature.

[66] Kabeya Y, Mizushima N, Ueno T, Yamamoto A, Kirisako T, Noda T, Kominami E, Ohsumi Y, Yoshimori T (2000). LC3, a mammalian homologue of yeast Apg8p, is localized in autophagosome membranes after processing. EMBO J.

[67] Kirisako T, Baba M, Ishihara N, Miyazawa K, Ohsumi M, Yoshimori T, Noda T, Ohsumi Y (1999). Formation process of autophagosome is traced with Apg8/Aut7p in yeast. J Cell Biol.

[68] Noda NN, Ohsumi Y, Inagaki F (2010). Atg8-family interacting motif crucial for selective autophagy. FEBS Lett.

[69] Pankiv S, Clausen TH, Lamark T, Brech A, Bruun JA, Outzen H, Overvatn A, Bjorkoy G, Johansen T (2007). p62/SQSTM1 binds directly to Atg8/LC3 to facilitate degradation of ubiquitinated protein aggregates by autophagy. J Biol Chem.

[70] Nakatogawa H, Ohbayashi S, Sakoh-Nakatogawa M, Kakuta S, Suzuki SW, Kirisako H, Kondo-Kakuta C, Noda NN, Yamamoto H, Ohsumi Y (2012). The autophagy-related protein kinase Atg1 interacts with the ubiquitin-like protein Atg8 via the Atg8 family interacting motif to facilitate autophagosome formation. J Biol Chem.

[71] Alemu EA, Lamark T, Torgersen KM, Birgisdottir AB, Larsen KB, Jain A, Olsvik H, Overvatn A, Kirkin V, Johansen T (2012). ATG8 family proteins act as scaffolds for assembly of the ULK complex: sequence requirements for LC3-interacting region (LIR) motifs. J Biol Chem.

[72] Suttangkakul A, Li F, Chung T, Vierstra RD (2011). The ATG1/ATG13 protein kinase complex is both a regulator and a target of autophagic recycling in *Arabidopsis*. Plant Cell.

[73] Koyama-Honda I, Itakura E, Fujiwara TK, Mizushima N (2013). Temporal analysis of recruitment of mammalian ATG proteins to the autophagosome formation site. Autophagy.

[74] Mizushima N, Yamamoto A, Hatano M, Kobayashi Y, Kabeya Y, Suzuki K, Tokuhisa T, Ohsumi Y, Yoshimori T (2001). Dissection of autophagosome formation using Apg5-deficient mouse embryonic stem cells. J Cell Biol.

[75] Suzuki H, Tabata K, Morita E, Kawasaki M, Kato R, Dobson RC, Yoshimori T, Wakatsuki S (2014). Structural basis of the autophagy-related LC3/Atg13 LIR complex: recognition and interaction mechanism. Structure.

[76] Stephan JS, Yeh YY, Ramachandran V, Deminoff SJ, Herman PK (2009). The Tor and PKA signaling pathways independently target the Atg1/Atg13 protein kinase complex to control autophagy. Proc Natl Acad Sci USA.

[77] Budovskaya YV, Stephan JS, Reggiori F, Klionsky DJ, Herman PK (2004). The Ras/cAMP-dependent protein kinase signaling pathway regulates an early step of the autophagy process in *Saccharomyces cerevisiae*. J Biol Chem.

[78] Schmelzle T, Beck T, Martin DE, Hall MN (2004). Activation of the RAS/cyclic AMP pathway suppresses a TOR deficiency in yeast. Mol Cell Biol.

[79] Noda T, Ohsumi Y (1998). Tor, a phosphatidylinositol kinase homologue, controls autophagy in yeast. J Biol Chem.

[80] Blommaart EF, Luiken JJ, Blommaart PJ, van Woerkom GM, Meijer AJ (1995). Phosphorylation of ribosomal protein S6 is inhibitory for autophagy in isolated rat hepatocytes. J Biol Chem.

[81] Kamada Y, Yoshino K, Kondo C, Kawamata T, Oshiro N, Yonezawa K, Ohsumi Y (2010). Tor directly controls the Atg1 kinase complex to regulate autophagy. Mol Cell Biol.

[82] Lee JW, Park S, Takahashi Y, Wang HG (2010). The association of AMPK with ULK1 regulates autophagy. PLoS One.

[83] Roach PJ (2011). AMPK -> ULK1 -> autophagy. Mol Cell Biol.

[84] Wang Z, Wilson WA, Fujino MA, Roach PJ (2001). Antagonistic controls of autophagy and glycogen accumulation by Snf1p, the yeast homolog of AMP-activated protein kinase, and the cyclin-dependent kinase Pho85p. Mol Cell Biol.

[85] Meley D, Bauvy C, Houben-Weerts JH, Dubbelhuis PF, Helmond MT, Codogno P, Meijer AJ (2006). AMP-activated protein kinase and the regulation of autophagic proteolysis. J Biol Chem.

[86] Inoki K, Zhu T, Guan KL (2003). TSC2 mediates cellular energy response to control cell growth and survival. Cell.

[87] Gwinn DM, Shackelford DB, Egan DF, Mihaylova MM, Mery A, Vasquez DS, Turk BE, Shaw RJ (2008). AMPK phosphorylation of raptor mediates a metabolic checkpoint. Mol Cell.

[88] Egan DF, Shackelford DB, Mihaylova MM, Gelino S, Kohnz RA, Mair W, Vasquez DS, Joshi A, Gwinn DM, Taylor R, Asara JM, Fitzpatrick J, Dillin A, Viollet B, Kundu M, Hansen M, Shaw RJ (2011). Phosphorylation of ULK1 (hATG1) by AMP-activated protein kinase connects energy sensing to mitophagy. Science.

[89] Kim J, Kundu M, Viollet B, Guan KL (2011). AMPK and mTOR regulate autophagy through direct phosphorylation of Ulk1. Nat Cell Biol.

[90] Shang L, Chen S, Du F, Li S, Zhao L, Wang X (2011). Nutrient starvation elicits an acute autophagic response mediated by Ulk1 dephosphorylation and its subsequent dissociation from AMPK. Proc Natl Acad Sci USA.

[91] Fimia GM, Stoykova A, Romagnoli A, Giunta L, Di Bartolomeo S, Nardacci R, Corazzari M, Fuoco C, Ucar A, Schwartz P, Gruss P, Piacentini M, Chowdhury K, Cecconi F (2007). Ambra1 regulates autophagy and development of the nervous system. Nature.

[92] Nazio F, Strappazzon F, Antonioli M, Bielli P, Cianfanelli V, Bordi M, Gretzmeier C, Dengjel J, Piacentini M, Fimia GM, Cecconi F (2013). mTOR inhibits autophagy by controlling ULK1 ubiquitylation, self-association and function through AMBRA1 and TRAF6. Nat Cell Biol.

[93] Itakura E, Mizushima N (2010). Characterization of autophagosome formation site by a hierarchical analysis of mammalian Atg proteins. Autophagy.

[94] Sekito T, Kawamata T, Ichikawa R, Suzuki K, Ohsumi Y (2009). Atg17 recruits Atg9 to organize the pre-autophagosomal structure. Genes Cells.

[95] Itakura E, Kishi-Itakura C, Koyama-Honda I, Mizushima N (2012). Structures containing Atg9A and the ULK1 complex independently target depolarized mitochondria at initial stages of Parkin-mediated mitophagy. J Cell Sci.

[96] Orsi A, Razi M, Dooley HC, Robinson D, Weston AE, Collinson LM, Tooze SA (2012). Dynamic and transient interactions of Atg9 with autophagosomes, but not membrane integration, are required for autophagy. Mol Biol Cell.

[97] Chang YY, Neufeld TP (2009). An Atg1/Atg13 complex with multiple roles in TOR-mediated autophagy regulation. Mol Biol Cell.

[98] Papinski D, Schuschnig M, Reiter W, Wilhelm L, Barnes CA, Maiolica A, Hansmann I, Pfaffenwimmer T, Kijanska M, Stoffel I, Lee SS, Brezovich A, Lou JH, Turk BE, Aebersold R, Ammerer G, Peter M, Kraft C (2014). Early steps in autophagy depend on direct phosphorylation of Atg9 by the Atg1 kinase. Mol Cell.

[99] Russell RC, Tian Y, Yuan H, Park HW, Chang YY, Kim J, Kim H, Neufeld TP, Dillin A, Guan KL (2013). ULK1 induces autophagy by phosphorylating Beclin-1 and activating VPS34 lipid kinase. Nat Cell Biol.

[100] Di Bartolomeo S, Corazzari M, Nazio F, Oliverio S, Lisi G, Antonioli M, Pagliarini V, Matteoni S, Fuoco C, Giunta L, D’Amelio M, Nardacci R, Romagnoli A, Piacentini M, Cecconi F, Fimia GM (2010). The dynamic interaction of AMBRA1 with the dynein motor complex regulates mammalian autophagy. J Cell Biol.

[101] Zheng J, Trafny EA, Knighton DR, Xuong NH, Taylor SS, Ten Eyck LF, Sowadski JM (1993). 2.2 A refined crystal structure of the catalytic subunit of cAMP-dependent protein kinase complexed with MnATP and a peptide inhibitor. Acta Crystallogr D Biol Crystallogr.

[102] Delano WL (2002). The PyMOL molecular graphics system.

